# Professionelle Teamarbeit und Kommunikation im Operationssaal – Eine narrative Übersicht

**DOI:** 10.1007/s00101-021-01027-1

**Published:** 2021-08-27

**Authors:** Anne Lammert, Markus Alb, Lena Huber, Frederic Jungbauer, Benedikt Kramer, Sonja Ludwig, Nicole Rotter, Lena Zaubitzer, Claudia Scherl

**Affiliations:** 1 Klinik für Hals-Nasen-Ohrenheilkunde, Kopf- und Halschirurgie, Universitätsmedizin Mannheim, Medizinische Fakultät Mannheim, Universität Heidelberg, Theodor-Kutzer-Ufer 1–3, 68167 Mannheim, Deutschland; 2 Abteilung Anästhesie und Schmerztherapie, Evangelisches Krankenhaus Bad Dürkheim, Bad Dürkheim, Deutschland

**Keywords:** OP-Management, Patientensicherheit, Konflikt, Time-out, Wirtschaftlichkeit, OR management, Patient safety, Conflict, Time out, Commercial efficiency

## Abstract

**Hintergrund:**

Ein Operationsteam ist ein für Störungen anfälliges Konstrukt aus hierarchisch und oft geschlechtlich differenten Mitgliedern, die unterschiedlichen Berufsgruppen angehören. Zieldiversitäten der einzelnen Akteure unter ökonomisch steigendem Leistungsdruck bergen Konfliktpotenzial im Arbeitsalltag. Dies kann negative Auswirkungen auf die Patientensicherheit und die wirtschaftliche Effizienz des Klinikbetriebs haben.

**Ziel:**

Ziel dieser Übersicht ist es, den Leser/die Leserin für die im operativen Klinikalltag bestehende Problematik zu sensibilisieren und ein Bewusstsein für die möglichen Lösungsansätze einer guten Kommunikationsstruktur und Teambildung sowie einer Moderation von außen durch ein Operationssaal(OP)-Management zu schaffen.

**Methoden:**

Narratives Review anhand einer Literaturrecherche relevanter Originalarbeiten und Expertenempfehlungen der Datenbanken *PubMed/MEDLINE*; Schlüsselworte u. a. Teamarbeit, Kommunikation, OP, Teambildung.

**Ergebnisse und Schlussfolgerung:**

Kommunikation und Teamarbeit im OP sind von immenser Bedeutung für die Patientensicherheit und die wirtschaftliche Entwicklung eines Klinikbetriebs. Verbesserungen in der Kommunikationsstruktur u. a. durch die konsequente Durchführung eines Team-Time-Out und eine „Moderation von außen“ (OP-Manager) bieten Lösungsansätze zur Vermeidung von Konfliktpotenzial im klinischen Alltag an.

## Abstract (en)

**Background:**

A team in the operating room (OR) is a hierarchically structured, gender-mixed group of people belonging to different professional categories. Disparities in the objectives of the different team members under economic pressure to perform, are sources of potential conflict in the daily work routine. This may have a negative impact on patient safety and commercial efficiency of hospital management.

**Objective:**

The aim of this summary is to sensitize the reader to the complex of problems in daily life in the OR and to increase awareness of possible approaches to solve the difficulties in an OR. Problem solutions might be approached by improvement of communication and team building.

**Methods:**

Narrative review of current literature and expert recommendations by a literature search in PubMed and Medline; keywords included teamwork, communication, operating room, team building.

**Results and conclusion:**

Communication and teamwork in the OR are of immense importance for patient safety and the economic development of a hospital. Improvements in communication structure, among other things due to the implementation of a team time out and moderation from outside (OR manager) offer solutions to avoid conflicts in everyday clinical practice.

## Einleitung

Im Operationssaal (OP) erfolgen medizinisch komplexe Prozeduren, die ein hohes Maß an Kompetenz und Verantwortungsbewusstsein vom durchführenden Team erfordern. Daneben sind OP-Kapazitäten eine der teuersten Klinikressourcen, die effektiv genutzt werden sollten. Das im OP tätige Personal unterliegt daher in besonderem Maße einer medizinischen und wirtschaftlichen Verantwortung. Ein OP-Team besteht üblicherweise aus hierarchisch und oft geschlechtlich differenten Mitgliedern, die außerdem unterschiedlichen Berufsgruppen angehören. Zieldiversitäten der einzelnen Akteure unter ökonomisch steigendem Leistungsdruck bergen Konfliktpotenzial im Arbeitsalltag. Dies kann negative Auswirkungen auf Patientensicherheit und wirtschaftliche Effizienz des Klinikbetriebs haben.

Durch eine gezielte Aufarbeitung und Zusammenfassung der bestehenden relevanten Literatur zum genannten Themenkomplex soll der Leser/die Leserin für die im operativen Klinikalltag bestehende Problematik sensibilisiert werden. Gleichzeitig soll ein Bewusstsein für die möglichen Lösungsansätze einer guten Kommunikationsstruktur und Teambildung im OP geschaffen werden.

## Methoden

Im Sinne eines narrativen Reviews wurde eine Literatursuche anhand der Datenbanken *PubMed* und *MEDLINE* durchgeführt. Es erfolgte eine Sichtung von Publikationen, die bis Mai 2021 veröffentlicht wurden. Die folgenden Schlüsselwörter wurden in erster Linie zur Suche verwendet: „teamwork“, „communication“, „operating room“, „teambuilding“, „OR“, „anaesthesiologist“, „nurse“, „surgeon“. Die geprüften Publikationen umfassen nichtsystematische Übersichtsarbeiten, Buchbeiträge, Fallstudien bis zu multizentrischen, prospektiven, randomisierten Fall-Kontroll-Studien. Publikationen, die in den Referenzlisten anderer Artikel zitiert wurden, wurden ebenfalls als mögliche Quellen betrachtet und in den Beurteilungsprozess aufgenommen.

## Ergebnisse und Diskussion

### Rollenverteilung im OP

Ein Team ist eine Gruppe von Personen, die gemeinsam an einer Aufgabe arbeiten. Die einzelnen Mitglieder eines Teams stehen dabei häufig in Abhängigkeit zueinander [4]. Ein OP ist besetzt von einem hierarchisch gegliederten, oft gemischtgeschlechtlichen Team, dem Mitarbeiter/innen unterschiedlicher Berufsgruppen angehören. Diese Durchmischung birgt Konfliktpotenzial, aber auch Chancen. Das Ziel eines OP-Teams ist die erfolgreiche, sichere Durchführung einer Operation. Die einzelnen Teammitglieder verfolgen darüber hinaus individuelle Ziele. Hoeper et al. zeigten in einer qualitativ-explorativen Studie, in der Chirurgen, Anästhesisten, OP- und Anästhesiepfleger interviewt wurden, Unterschiede in der Bewertung der individuellen Ziele [7]. Für die Chirurgen waren wirtschaftliche Effizienz und medizinische Qualität vorrangig, für Anästhesiologen Mitarbeiterzufriedenheit, gefolgt von der wirtschaftlichen Effizienz. Hierbei zeigte sich mit zunehmender Seniorität (Ober‑/Chefärzte/-innen vs. Assistenten/-innen) eine Zunahme der Bedeutung von Qualität und Wirtschaftlichkeit im Vergleich zur Mitarbeiterzufriedenheit. Für die Pflegenden stand die Mitarbeiterzufriedenheit an erster Stelle. Dies zeigt, dass die beteiligten Berufsgruppen die Zieldimensionen eines Klinikbetriebs wie medizinische Qualität, Wirtschaftlichkeit, Mitarbeiterzufriedenheit, Patientenzufriedenheit, Lehre und Forschung unterschiedlich bewerten. Hieraus ergeben sich Konflikte. Der klinische Alltag bringt oft eine ständig wechselnde Teamzusammensetzung mit sich. Dadurch wird eine gegenseitige Anpassung innerhalb des Teams zusätzlich behindert. Das erhöht das Konfliktpotenzial weiter. Ein möglicher Lösungsansatz kann die gegenseitige Kommunikation der Zielbewertungen sein, um Managementsysteme zu verbessern und Konfliktpotenziale zu reduzieren. Knappe Finanzmittel und ökonomischer Erfolgsdruck ziehen eine Leistungsverdichtung für alle im OP tätigen Berufsgruppen nach sich. Der Arbeitsplatz „Krankenhaus“ wird dadurch zunehmend unattraktiv. Kliniken leiden unter den Folgen der Umorientierung von Mitarbeitern/-innen der Pflege aus den Krankenhäusern heraus. Diese Situation wird kurzfristig durch engagiertes Personal kompensiert; mittelfristig werden Resignation und eine weitere „Abwanderung“ von Mitarbeitern/-innen beobachtet. Dies kann langfristig die Existenz einer Klinik gefährden [6]. Die eintretende „Mangelverwaltung“ führt vielerorts zu einer „Abwärtsspirale“. Maßnahmen zur besseren Mitarbeiterorientierung stellen notwendige Investitionen dar. Teambasiertes Arbeiten ist im medizinischen Bereich im Vergleich zu anderen Dienstleistungsbereichen unzureichend ausgebildet. Es fehlt an Kenntnissen über die Komplexität der Teambildung zwischen unterschiedlichen Berufsgruppen, wie es in einem OP-Team der Fall ist. Dadurch besteht wenig Verständnis für die Zerbrechlichkeit der kollegialen Bande der Interdisziplinarität, die in der Zusammenarbeit im OP so wertvoll sind. Diese spielen jedoch gerade hier eine zentrale Rolle, um Patientensicherheit und ökonomische Effizienz zu leisten [4]. Ein Kompetenzmix und divergierende Individualziele können nur durch gegenseitiges Verständnis, zwischenmenschliche Kommunikation und Moderation von außen positiv beeinflusst werden [6, 7]. Dafür existieren im Gegensatz zu anderen Dienstleistungsunternehmen in vielen Kliniken weder ein Bewusstsein noch die Ressourcen, diesem Problem zu begegnen.

Gemäß einer Untersuchung von Leach et al. sind sich die Mitglieder eines OP-Teams in einer Sache einig: Das Arbeitsumfeld ist der am häufigsten genannte beeinflussende Faktor, und die Person, die dieses Umfeld am meisten zu beeinflussen vermag, sei der Chirurg/die Chirurgin [13]. Effektive Kommunikation ist dabei neben klinischer Expertise ein Grundstein der Arbeit im OP [25]. Ein guter Chirurg sollte daher hinterfragen: Bin ich ein guter Teamplayer?

Unter einer Operation aufkommende Konflikte sollten möglichst sofort durch klare Kommunikation geklärt werden. Da nicht selten eine sofortige Klärung unter einer Operation nicht möglich ist, muss sichergestellt sein, dass es in diesem Falle die Möglichkeit einer Nachbesprechung gibt [12].

#### Rollenverteilung zwischen Chirurg und Anästhesist

Unterschiedliche individuelle Ziele können im klinischen Alltag Auseinandersetzungen zwischen Chirurg und Anästhesist bedingen [7]. Nach Jones et al. sind es oft Chirurgen, von denen ein Streit ausgeht. Die „Streithierarchie“ wird durch die Reihenfolge „Chirurg, Assistenzchirurg, Anästhesist, OP-Pfleger“ beschrieben. Dabei seien es v. a. Männer, die zu Diskussionen neigen, während eine Geschlechterdurchmischung deeskalierend wirkt [9]. Dennoch machten Streits nur einen Bruchteil der Kommunikation aus – nur 2,8 % wurden als „Konfliktfälle“ eingestuft. In 80 % der „Konfliktfälle“ begann die Person den Streit, die einen höheren Status hatte. In zwei Dritteln der Fälle handelte es sich um den Chirurgen [9].

Chirurgen werden neben ihren technischen Fähigkeiten auch nach Merkmalen wie Kommunikationsfertigkeiten/Teamfähigkeit beurteilt [24, 32]. Wauben et al. stellten fest, dass Chirurgen ihre eigenen Kommunikationsfähigkeiten besser einstufen, als die anderen Teammitglieder diese empfinden [31]. Mehr als die Hälfte der befragten Anästhesisten bemängelten, dass ihre chirurgischen Kollegen u. a. nicht darüber informieren, wenn die Operation nicht planmäßig verläuft. Die Kommunikation im OP wird von 72 % der OP-Pflegenden und 44 % der Anästhesisten bemängelt, jedoch von nur 6 % der Chirurgen - d.h., Chirurgen sind im Vergleich mit der Qualität der Kommunikation zufriedener. Sie merken demnach nicht, dass sie von den anderen Berufsgruppen schlecht bewertet werden [31].

Typische Formen der schlechten Kommunikation sind aggressive Wortwahl und Wortlaut, Unhöflichkeit und Grobheiten wie Schreien und Beschimpfen. Katz et al. setzten anästhesiologische Assistenzärzte/-innen in einem simulierten Szenario der Situation einer verstärken intraoperativen Blutung, vergleichend einem „normalen“ oder einem „groben, unhöflichen“ Arbeitsumfeld, aus. Dieses wurde durch den Chirurgen verkörpert, der einmal einen „normalen“ und einmal einen „groben, unhöflichen“ Umgangston wählte. Obwohl die beobachteten jungen Anästhesisten/-innen in einer Selbstbewertung ihre Leistung bezüglich (nicht)technischer Fähigkeiten wie Diagnosestellung und Wachsamkeit unter beiden Bedingungsumfeldern gleich einschätzten, hatte der „grobe Umgangston“ negativen Einfluss auf ihre Leistungsfähigkeit [11]. Interpersonelle Kommunikation in Stresssituationen sollte demnach zur medizinischen Ausbildung gehören. Chirurgen tragen entsprechend neben ihrer manuellen Tätigkeit im OP auch im Wesentlichen die Verantwortung dafür, dass effektives Teamwork entsteht [30].

Chirurgen/-innen haben ein intrinsisches Interesse, ihre Patienten zeitnah behandeln/operieren zu können. Im Jahr 2010 wurden die Kosten einer OP-Minute in Deutschland, abhängig u. a. von der Berechnungsgrundlage, mit 10 bis 120 € veranschlagt [2]. Zwar wird in europäischen Gesundheitssystemen die OP-Minute nicht den operativen Disziplinen in Rechnung gestellt, jedoch unterliegen chirurgische Disziplinen dadurch einer strengen Kontrolle ihrer Leistungszahlen durch die Klinikleitungen. Da die teure Ressource „OP-Kapazität“ begrenzt ist, entsteht eine Konkurrenzsituation zwischen den operativen Disziplinen. Dieser Druck wird durch den Chirurgen an das Gesamt-OP-Team weitergegeben. Zusätzlich tendieren Chirurgen folglich dazu, ihre Operationszeit zu unterschätzen, um die Ressourcenknappheit keinesfalls unterzubelegen. Dies führt häufig zu einer Überlastung der OP-Kapazitäten [3, 7, 23]. Diese Vorgehensweise steht im Gegensatz zum Fokus der Mitarbeiterzufriedenheit [7], denn es entstehen hetzende Situationen und Überstunden. Anästhesiologen hingegen müssen sich aufgrund der beschriebenen Konkurrenzsituation „zwischen den chirurgischen Disziplinen zerreißen“, werden jedoch am Ende weniger hinsichtlich der Quantität der abgeleisteten OP-Punkte bewertet. Anästhesiologische Abteilungen werden von außen häufig v. a. hinsichtlich ihrer Kostenentwicklung, weniger hinsichtlich ihrer Leistungsentwicklung beurteilt.

#### Rollenverteilung zwischen ärztlichen und pflegenden Mitgliedern des OP-Teams

Es wurde bereits vorher erwähnt, dass die Mitarbeiterzufriedenheit für die pflegenden Mitglieder des OP-Teams im Fokus steht. Außerdem unterscheiden sich die Mitarbeiter/-innen der Pflege in dieser Priorisierung bezüglich ihrer hierarchischen Stufe unwesentlich. Die OP- und Anästhesiepflege bilden unabhängig von ihrer hierarchischen Stellung eher eine Einheit im Vergleich zu den ärztlichen Akteuren. Deren Priorisierung verschiebt sich im Laufe ihrer Karriere eher in die Richtung der wirtschaftlichen Effizienz und medizinischen Qualität [7]. Hier ist zu beobachten, dass sich diesbezüglich Chirurgen/-innen und Anästhesisten/-innen eher gleichen. In beiden ärztlichen Gruppen verschiebt sich der Fokus der täglichen Arbeit von der Mitarbeiterzufriedenheit (bei Weiterbildungsassistenten/-innen) hin zur Fokussierung auf Wirtschaftlichkeit und Effizienz (Ober- und Chefärzte/-innen). Dieser Effekt ist im Übrigen bei Anästhesisten stärker ausgeprägt als bei Chirurgen. Auch sei hervorgehoben, dass Anästhesisten und die Teammitglieder der Pflege mit ihrem Fokus auf die Mitarbeiterzufriedenheit den chirurgisch Tätigen gegenüberstehen [7]. In näher ausgeführten Interviews unterstreichen die Mitarbeiter der Pflege ihre Beschreibung der Mitarbeiterzufriedenheit u. a. durch gute Kommunikation zwischen den Berufsgruppen und gegenseitige Wertschätzung. Mitarbeiter/-innen der Pflege definieren ein OP-Team als Einheit, während sowohl Chirurgen/-innen als auch Anästhesisten/-innen unabhängige Untergruppen sehen [7, 28]. Insbesondere Chirurgen werden im Rahmen ihrer Ausbildung zu „Individualisten erzogen“ [1]. Dies beginnt mit dem auf das Individuum bezogenen Medizinstudium. Auch später stehen in der medizinischen Ausbildung Ziele wie „Teambildung“ eher im Hintergrund.

Teammitglieder der Pflege bemerken in einer Vielzahl von Fällen unangemessenes Verhalten bei Tätigkeitsmerkmalen der beteiligten Berufsgruppen Chirurg und Anästhesist. 72 % benennen nach Wauben et al. hinsichtlich der „allgemeinen Zufriedenheit mit Kommunikation und Teamwork im OP“, ein unangemessenes Verhalten beobachtet zu haben [9, 12, 31]. Kommunikationsfähigkeit und Teamwork sind neben der Fähigkeit zur Stressbewältigung die wichtigsten „nichttechnischen“ Schlüsselfähigkeiten, die in Interviews sowohl von OP-Pflegern/-innen selbst als auch von Chirurgen/-innen gleichermaßen benannt und gegenseitig erwartet werden [20, 21].

Gerade in der heutigen Zeit des „Pflegemangels“ wird durch Maßnahmen wie das spontane Überführen von Pflegepersonal von einer in die andere chirurgische Fachdisziplin oder das immer häufiger notwendige Hinzuziehen von Pflegepersonal über Fachpflegevermittlungen („nurse to rent“®) versucht, dem Mangel Herr zu werden. Dadurch kommt es zur Bildung von „Ad-hoc-Teams“. Diese zeichnen sich dadurch aus, dass sie nach Bedarf und nur für kurze Zeit gebildet werden, um eine bestimmte Aufgabe anzugehen. Sie lösen sich anschließend rasch wieder auf [8]. In der Wirtschaft wird bei dieser Art der Teambildung als besonders vorteilhaft gesehen, dass sich keine festen Muster der Teamarbeit ausbilden können, z. B. keine Festlegung bestimmter Personen auf bestimmte Anforderungen. Im klinischen Alltag werden bei dieser Form der Teambildung zeitliche Verzögerungen und der Verlust von Expertise und Identifikation der OP-Teams unterschiedlicher chirurgischer Fächer beobachtet. Es bildet sich im klinischen Alltag ab, dass die oben beschriebene Form der „Pflegemangelverwaltung“ durch u. a. die Mobilisation der verbleibenden pflegenden Mitarbeiter aus ihren vertrauten Fachbereichen heraus, eine Form der Resignation auslöst, die eine weitere Verstärkung der Abwanderung qualifizierten Personals aus den Krankenhäusern hinaus nach sich ziehen könnte [10].

### Folgen vernachlässigten Teamworks

Von Ligand et al. wurde gezeigt, dass ein Drittel der Gesprächssituationen innerhalb eines OP-Teams fehlerhaft ist. In wiederum einem Drittel der fehlerhaften Kommunikationssituationen kam es zu Effekten auf Systemprozesse (u. a. zwischenmenschliche Spannungen, Ressourcenverschwendung) [14]. Folgen wie Spannungen im Team und die Unterbrechung der Routine durch provisorische Übergangslösungen können die Sicherheit eines Patienten potenziell gefährden.

Nach Mazzocco et al. erhöht schlechte Kommunikation in einem OP-Team die Komplikationsrate und die Gefahr einer Operation mit letalem Ausgang fast um den Faktor 5 [12, 19]. Die niederschmetternde Kernaussage der Gruppe lautete abschließend: Wenn die Akteure im OP nur selten „Team-Verhaltensweisen“ aufweisen, ist es für den Patienten eher möglich, den Tod oder eine größere Komplikation zu erleiden [19].

In einer Übersichtsarbeit von Manser [16] wurden wissenschaftliche Hauptgebiete identifiziert, die den Zusammenhang zwischen Teamarbeit und Patientensicherheit untermauern:

1. Studien, die untersuchen, welche Faktoren dazu beisteuern, dass kritische oder unerwünschte Ereignisse eintreten, konnten zeigen, dass Teamwork eine wichtige Rolle sowohl in der Entstehung als auch in der Prävention derselben spielt.
2. Forschungen, die auf die Auffassung von „Teamwork“ bei Leistungserbringern im Gesundheitswesen fokussieren, zeigen,
  dass die Auffassung der Belegschaft von Teamwork und ihre Haltung hinsichtlich sicherheitsrelevantem Teamverhalten mit Qualität und Sicherheit in der Patientenversorgung in Beziehung stehen,
  dass die Auffassung von Teamwork und Führungsstil am Arbeitsplatz mit der Mitarbeiterzufriedenheit assoziiert ist, was die Fähigkeit z. B. eines OP-Teams stärkt, Patientensicherheit zu gewährleisten.
3. Beobachtungsstudien, die Teamwork-Verhalten mit hoher klinischer Leistungsfähigkeit in Beziehung bringen, haben Strukturen in Kommunikation, Koordination und Führungsverhalten identifiziert, die effektives Teamwork unterstützen.

Demnach ziehen schlechte Kommunikation/schlechtes Teamwork kritische Ereignisse nach sich, die die Qualität und Sicherheit verschlechtern und die Mitarbeiterzufriedenheit und Leistungsfähigkeit schwächen [15].

### Werkzeuge zur Verbesserung von Kommunikation und Teamwork im OP

**Figure 1:**
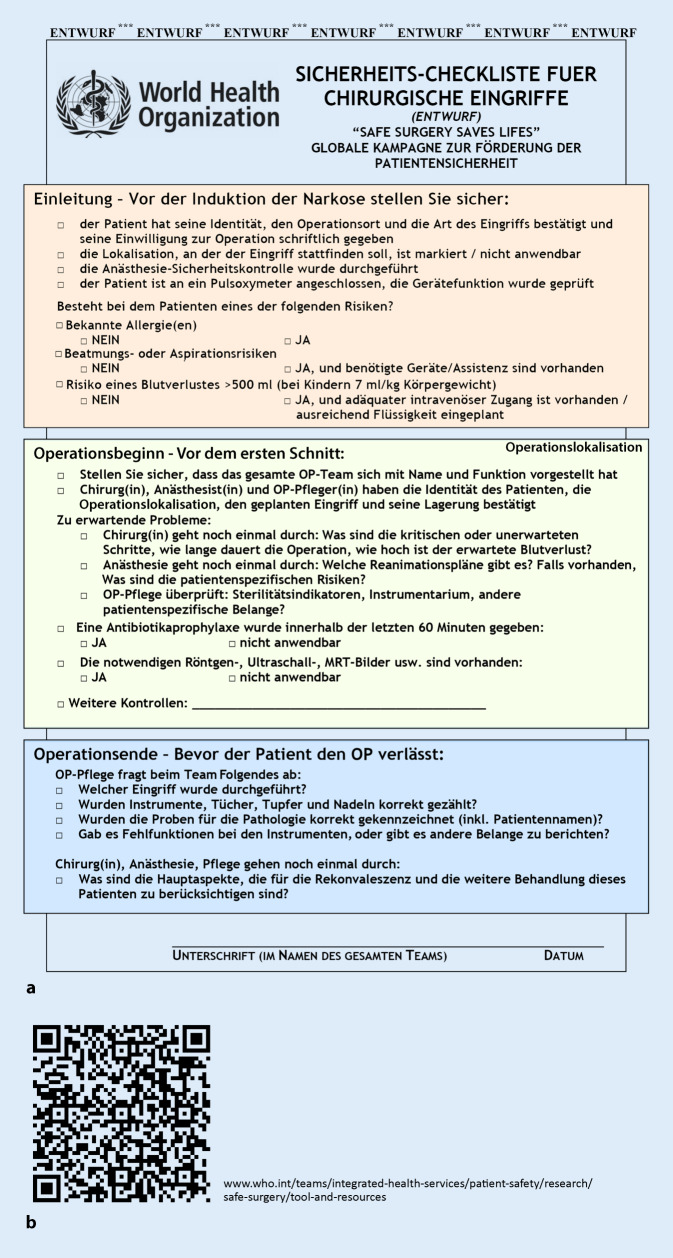

**Figure 2:**
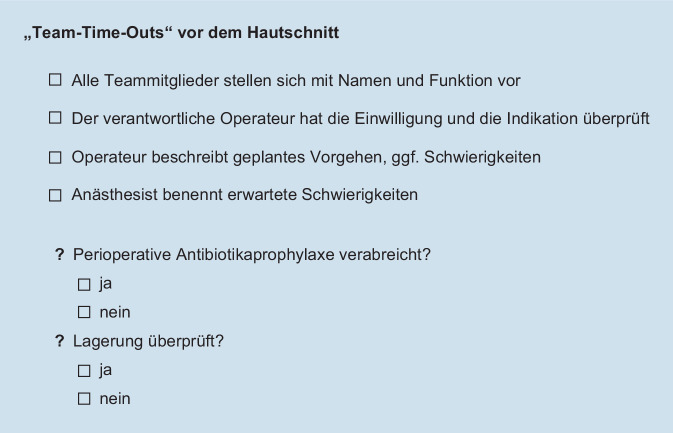

Je nach Komplexität der chirurgischen Prozedur und des zu behandelnden Patienten kommt es in 3–12 % der Fälle zu unerwünschten Ereignissen, die häufig auf kommunikativen Problemen im Team beruhen [13]. Bereits vor über 15 Jahren beschäftigte sich die Weltgesundheitsorganisation (WHO) mit der Frage, wie die Sicherheit unserer Patienten im OP verbessert werden könne. Vorbildern v. a. aus der Luftfahrt folgend wurde eine Kontrollliste (Surgical Safety Checklist) implementiert, deren Einsatz die Weltgesundheitsorganisation (WHO) seit 2008 empfiehlt (Abb. 1) und die je nach den Bedürfnissen eines Teams angepasst werden kann (Abb. 2). Eine Vielzahl von Studien konnte die Wirksamkeit derselben hinsichtlich der Senkung der perioperativen Mortalität/Morbidität belegen [19, 25, 32]. Ihr Effekt wurde in 8 Ländern mit unterschiedlichen sozioökonomischen Strukturen evaluiert. Dabei wurden rund 4000 Patienten, jeweils vor und nach der Implementierung der Checkliste, bewertet. Die Rate schwerer Komplikationen fiel von 11 auf 7 %, die perioperative Mortalität bei großen Operationen von 1,5 auf 0,8 % [27]. Perioperativ tätiges ärztliches und pflegendes Personal ist mit einer Vielzahl von Theorien, Modellen und Methoden zur Optimierung der Patientensicherheit konfrontiert. Die Herausforderung besteht daher darin, all diese zu einem funktionierenden Risikomanagementkonzept zusammenzuführen, das die jeweiligen Stärken und Schwächen der einzelnen Elemente kennt und berücksichtigt [22]. Jedes Mitglied eines operativen Teams trägt somit zum Gelingen der Integrierung z. B. der Surgical Safety Checklist bei.

Das sog. Team-Time-Out nimmt hier eine zentrale Rolle ein. Dies hat den Effekt, dass für das chirurgische Resultat des Patienten essenzielle Vorgänge überprüft und ggf. korrigiert werden. Dieses verlangt vor dem Beginn der Operation („vor Schnitt“) das „Innehalten des gesamten operativen Teams“ – was so tiefgründig und bedeutend klingt, hat den simplen, jedoch entscheidenden Effekt, für das chirurgische Resultat des Patienten ganz essenzielle Vorgänge zu planen, zu überprüfen und ggf. zu korrigieren – die Patientenidentität wird abschließend überprüft, die durchzuführende Prozedur und die Seite der Inzision. Zum Beispiel werden die Notwendigkeit und der Zeitpunkt einer bestenfalls präoperativ zu verabreichenden Antibiotikaprophylaxe geklärt bzw. überprüft. Darüber hinaus erfolgt eine Vorstellung aller anwesenden Teammitglieder, was Unsicherheiten im Umgang miteinander vermindern und die Zusammenarbeit verbessern kann. Ein chirurgischer Oberarzt, der im Rahmen des Team-Time-Out erfährt, dass die anästhesiologische Assistenzärztin im ersten Jahr ist, sich nun seit 3 Wochen in ihrer Rotation im Kopf-Hals-chirurgischen OP befindet und mit dem Atemwegsmanagement bei der Anlage eines stabilen Tracheostomas noch keinerlei Erfahrung hat, wird seine Art der Kommunikation intraoperativ hoffentlich an diesen Erfahrungsstand der Kollegin anpassen – eine Form der zwischenmenschlichen, kollegialen Interaktion, die man von jedem Akteur eines OP-Teams gegenüber den anderen Teammitgliedern erwarten sollte. Leider sieht der klinische Alltag erfahrungsgemäß oft anders aus: Zeit- und Leistungsdruck untergraben nicht selten die Kommunikation untereinander, was unseren Sinn für die Relevanz des Team-Time-Out nur stärker schärfen sollte. Das Team-Time-Out zwingt zur Kommunikation relevanter Fakten an das chirurgische Team und hat einen stark mannschaftsbildenden Effekt.

Durch Urbach et al. wurde in Frage gestellt, ob für die Wirksamkeit der WHO-Checkliste – und damit auch des enthaltenen Team-Time-Out – ausreichend Evidenz bestehe [29]. Die öffentliche Diskussion in Form von geposteten Reaktionen im Internet ließ nicht lange auf sich warten und der Tenor sowohl von Kommentaren anästhesiologischer als auch chirurgischer Seite ist, dass Checklisten einen wahrhaftigen und bedeutenden Nutzen im klinischen Alltag haben, dieser jedoch nur durch das Engagement von OP-Pflegenden, Chirurgen, Anästhesisten und OP-Managern belebt werden kann. Auch Urbach et al. betonten die Kernbedeutung von Teambildung und Kommunikation im OP [29].

Ein Lösungsansatz zur Bändigung des komplexen Arbeitsumfelds „OP“ durch eine „Moderation von außen“ besteht durch den Einsatz von „OP-Managern“. Diese sollen sich um die Sicherstellung einer medizinischen Versorgungsqualität bei gleichzeitiger Einhaltung ökonomischer Rahmenbedingungen bemühen. Doch wer ist geeignet, diese Aufgabe zu meistern – ein Anästhesist, ein Chirurg, die OP-Pflege – oder jemand, der „unparteiisch“ agieren kann oder muss?

Laut Schüpfer und Bauer zeichnet sich ein OP-Manager durch Ergebnisqualität aus. Er ist verantwortlich für die Leistungsfähigkeit des Unternehmens „Krankenhaus“ und definiert sich durch die Grundaufgaben a) für Ziele sorgen, b) entscheiden, c) kontrollieren (messen und beurteilen), d) organisieren (Ablauf- und Aufbauorganisation) und e) Menschen entwickeln (fördern). OP-Manager sollen mit dem OP-Bereich einen hinsichtlich der Erlös-Kosten-Struktur bedeutendsten Leistungsbereich der stationären Versorgung (mit)führen, organisieren und kontrollieren. Kliniken können sich daher ein unprofessionelles OP-Management nicht leisten. Der Auswahl eines geeigneten OP-Managers kommt eine zentrale unternehmerische Bedeutung zu. Es drängt sich der Gedanke auf, dass keiner der üblichen Akteure eines OP-Teams (Chirurg, Anästhesist, OP-/Anästhesiepflege) kapazitativ oder qualitativ die Aufgaben eines „OP-Managers“ erfüllen kann, auch wenn es aus unterschiedlichen Gründen immer wieder so praktiziert wird. Denn bei den Aufgaben eines OP-Managers handelt es sich nicht um eine Zusatzaufgabe, sondern um einen 100 %-Job. Ein OP-Manager muss zur Bewältigung der ihm gestellten Aufgaben zu eigenständigem Handeln legitimiert sein. Schon aufgrund der oben genannten differenten Zielsetzungen genügt ein etablierter Kliniker aus seinem klinischen Rollenverständnis heraus nicht per se oder eben gerade nicht dem Anforderungsprofil eines „OP-Managers“ [26]. Nach Marjamaa et al. ist es am ehesten historisch begründet, dass die Rollenaufteilung bezüglich verwaltender (Management‑)Aufgaben im sensiblen Arbeitsumfeld des OP häufig unklar ist [18]. Jedoch ist es von entscheidender Bedeutung, dass Stellung und Autorität der eingesetzten Person be- und anerkannt sind. Auf diese Weise können Konflikte schnell gelöst und Ressourcenzuweisungen effizient erfolgen [14]. Eine als OP-Manager eingesetzte Person sollte neben unternehmerischen Fähigkeiten über Führungsqualitäten und ein hohes Maß an emotionaler Intelligenz verfügen, wobei der Beruf/das Fachgebiet der Person von nachrangiger Bedeutung erscheinen [17, 18]. In einer Untersuchung von Gebhard et al. ergab eine Personalbefragung in einem chirurgischen Zentral-OP den Wunsch nach der Etablierung einer zentralen OP-Organisation, um Kommunikationsprobleme und OP-Planungsabläufe zu verbessern. Entgegen einer alten Überzeugung wurde kein einzelner OP-Manager ernannt, sondern ein Team „OP-Organisation“, bestehend aus je einem Vertreter der Chirurgie, der Anästhesie, der OP-Pflege und der Anästhesiepflege [5]. Es handelt sich dabei um einen Trend, der auch an unserem Universitätsklinikum bereits seit geraumer Zeit implementiert wurde. Es stellt sich somit heraus, dass auch vonseiten des Gesamt-OP-Teams ein starker Wunsch nach im Gedanken gelebter Interdisziplinarität besteht – ganz im Sinne der seit 2000 lautenden europäischen Losung „In Vielfalt geeint“.

Bei allem wirtschaftlichen Druck und der Diskussion über die Rollenverteilung im OP wird das primäre Ziel des OP-Teams im klinischen Alltag selten erwähnt: die sichere, erfolgreiche Durchführung einer Operation eines Patienten. Die Erklärung dafür ist glücklicherweise einfach: Dieses Ziel ist in allen beteiligten Berufsgruppen so verinnerlicht, dass hierüber nicht gesprochen werden muss [4].

## Fazit

- Ein OP-Team ist ein personell und hierarchisch durchmischtes Konstrukt mit differenten Teilinteressen und daher für innere und äußere Störungen anfällig.
- Die Individualziele von Chirurgen, Anästhesisten und pflegenden Mitgliedern eines OP-Teams bergen Konfliktpotenzial, das durch Leistungsverdichtung verschärft wird.
- Lösungsansätze zur Reduktion von Konflikten können Verbesserungen in der Kommunikationsstruktur und eine „Moderation von außen“ (OP-Manager) sein.
- Kommunikation und Teamarbeit im OP sind von immenser Bedeutung für die Patientensicherheit und die wirtschaftliche Entwicklung eines Klinikbetriebs.
